# 2-Methyl­piperazinediium tetra­chlorido­zincate(II)

**DOI:** 10.1107/S1600536810012547

**Published:** 2010-04-10

**Authors:** Ming Yin, Shao-Tong Wu

**Affiliations:** a School of Materials Science and Engineering, Jiangsu University of Science and Technology, Zhenjiang 212003, People’s Republic of China

## Abstract

The asymmetric unit of the title compound, (C_5_H_14_N_2_)[ZnCl_4_], consists of a diprotonated 2-methyl­piperazine cation and a tetra­chloridozincate anion. The Zn^II^ ion is in a slightly distorted tetra­hedral coordination environment. The six-membered piperazine ring adopts a chair conformation. The crystal structure is stabilized by inter­molecular N—H⋯Cl hydrogen bonds.

## Related literature

For ferroelectricity in coordination polymers, see: Fu *et al.* (2007 ▶). For nonlinear optical second harmonic generation induced by coordination polymers, see: Qu *et al.* (2003 ▶). For transition-metal complexes of (*R*)-2-methyl­piperazine, see: Ye *et al.* (2009 ▶).
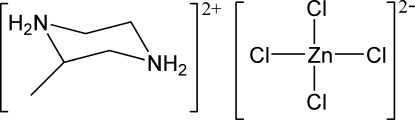


## Experimental

### 

#### Crystal data

(C_5_H_14_N_2_)[ZnCl_4_]
*M* *_r_* = 309.35
Monoclinic, 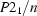

*a* = 8.4183 (17) Å
*b* = 14.939 (3) Å
*c* = 9.830 (2) Å
β = 90.35 (3)°
*V* = 1236.3 (4) Å^3^
*Z* = 4
Mo *K*α radiation
μ = 2.81 mm^−1^
*T* = 291 K
0.35 × 0.25 × 0.15 mm

#### Data collection

Rigaku SCXmini CCD diffractometer
Absorption correction: multi-scan (*CrystalClear*; Rigaku, 2005 ▶) *T*_min_ = 0.440, *T*_max_ = 0.678
11203 measured reflections
2423 independent reflections
2112 reflections with *I* > 2σ(*I*)
*R*_int_ = 0.045

#### Refinement

*R*[*F* ^2^ > 2σ(*F* ^2^)] = 0.037
*wR*(*F* ^2^) = 0.088
*S* = 1.11
2423 reflections
110 parameters
H-atom parameters constrained
Δρ_max_ = 0.67 e Å^−3^
Δρ_min_ = −0.45 e Å^−3^

### 

Data collection: *CrystalClear* (Rigaku, 2005 ▶); cell refinement: *CrystalClear*; data reduction: *CrystalClear*; program(s) used to solve structure: *SHELXS97* (Sheldrick, 2008 ▶); program(s) used to refine structure: *SHELXL97* (Sheldrick, 2008 ▶); molecular graphics: *SHELXTL* (Sheldrick, 2008 ▶) and *DIAMOND* (Brandenburg, 1999 ▶); software used to prepare material for publication: *SHELXTL*.

## Supplementary Material

Crystal structure: contains datablocks I, global. DOI: 10.1107/S1600536810012547/hy2296sup1.cif

Structure factors: contains datablocks I. DOI: 10.1107/S1600536810012547/hy2296Isup2.hkl

Additional supplementary materials:  crystallographic information; 3D view; checkCIF report

## Figures and Tables

**Table 1:** Hydrogen-bond geometry (Å, °)

*D*—H⋯*A*	*D*—H	H⋯*A*	*D*⋯*A*	*D*—H⋯*A*
N1—H1*A*⋯Cl2^i^	0.90	2.48	3.346 (3)	161
N1—H1*B*⋯Cl3^ii^	0.90	2.55	3.284 (3)	140
N1—H1*B*⋯Cl1^ii^	0.90	2.72	3.322 (3)	125
N2—H2*A*⋯Cl4	0.90	2.25	3.150 (3)	174
N2—H2*B*⋯Cl2^iii^	0.90	2.48	3.199 (3)	137
N2—H2*B*⋯Cl3^iii^	0.90	2.77	3.444 (3)	133

Symmetry codes: (i) 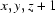; (ii) 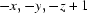; (iii) 
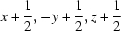
.

**Fig. 1.**
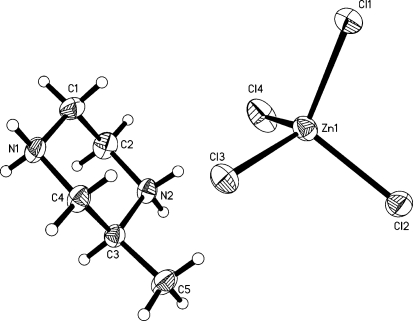
The asymmetric unit of the title compound. Displacement ellipsoids are drawn at the 30% probability level.

**Fig. 2.**
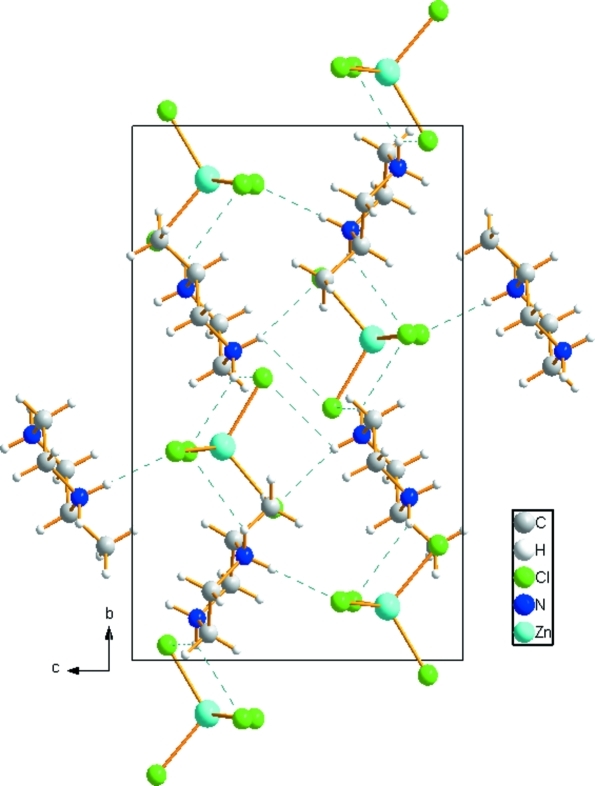
The crystal packing viewed along the a axis. Hydrogen bonds are drawn as dashed lines.

## supplementary crystallographic information

### Comment

The existence of a chiral center in an organic ligand is very important for the construction noncentrosymmetric or chiral coordination polymers that exhibit desirable physical properties such as ferroelectricity (Fu *et al.*, 2007) and nonlinear optical second harmonic generation (Qu *et al.*, 2003). Chiral (*R*)-2-methylpiperazine has shown tremendous scope in the synthesis of transition-metal complexes (Ye *et al.*, 2009). The construction of new members of this family of ligands is an important direction in the development of modern coordination chemistry. We report here the crystal structure of the title compound.

The asymmetric unit of the title compound consists of a diprotonated (±)-2-methylpiperazine cation and a tetrachloridozinc anion with the Zn^II^ ion in a slightly distorted tetrahedral coordination environment (Fig. 1). The 6-membered ring of piperazine adopts a chair conformation. The crystal structure is stabilized by intermolecular N—H···Cl hydrogen bonds (Table 1). The hydrogen bonds form a three-dimensional network (Fig. 2).

### Experimental

A mixture of (±)-2-methylpiperazine (1 mmol, 0.100 g), ZnCl_2_ (1 mmol, 0.136 g) and 10% aqueous HCl (6 ml) was dissolved in 30 ml water by heating to 353 K (10 min), forming a clear solution. The reaction mixture was cooled slowly to room temperature and crystals of the title compound formed after 6 d.

### Refinement

H atoms were placed in calculated positions and refined using a riding model, with C—H = 0.98 (CH), 0.97 (CH_2_) and 0.96 (CH_3_) Å, N—H = 0.90 Å and with *U*_iso_(H) = 1.2(1.5 for methyl)*U*_eq_(C, N).

### Crystal data

**Table:**

(C_5_H_14_N_2_)[ZnCl_4_]	*F*(000) = 624
*M**_r_* = 309.35	*D*_x_ = 1.662 Mg m^−^^3^
Monoclinic, *P*2_1_/*n*	Mo *K*α radiation, λ = 0.71073 Å
Hall symbol: -P 2yn	Cell parameters from 2112 reflections
*a* = 8.4183 (17) Å	θ = 3.2–26.0°
*b* = 14.939 (3) Å	µ = 2.81 mm^−^^1^
*c* = 9.830 (2) Å	*T* = 291 K
β = 90.35 (3)°	Block, colorless
*V* = 1236.3 (4) Å^3^	0.35 × 0.25 × 0.15 mm
*Z* = 4	

### Data collection

**Table:**

Rigaku SCXmini CCD diffractometer	2423 independent reflections
Radiation source: fine-focus sealed tube	2112 reflections with *I* > 2σ(*I*)
graphite	*R*_int_ = 0.045
Detector resolution: 13.6612 pixels mm^-1^	θ_max_ = 26.0°, θ_min_ = 3.2°
ω scans	*h* = −10→10
Absorption correction: multi-scan (*CrystalClear*; Rigaku, 2005)	*k* = −18→18
*T*_min_ = 0.440, *T*_max_ = 0.678	*l* = −12→12
11203 measured reflections	

### Refinement

**Table:**

Refinement on *F*^2^	Primary atom site location: structure-invariant direct methods
Least-squares matrix: full	Secondary atom site location: difference Fourier map
*R*[*F*^2^ > 2σ(*F*^2^)] = 0.037	Hydrogen site location: inferred from neighbouring sites
*wR*(*F*^2^) = 0.088	H-atom parameters constrained
*S* = 1.11	*w* = 1/[σ^2^(*F*_o_^2^) + (0.0275*P*)^2^ + 1.3805*P*] where *P* = (*F*_o_^2^ + 2*F*_c_^2^)/3
2423 reflections	(Δ/σ)_max_ < 0.001
110 parameters	Δρ_max_ = 0.67 e Å^−^^3^
0 restraints	Δρ_min_ = −0.45 e Å^−^^3^

### Fractional atomic coordinates and isotropic or equivalent isotropic displacement parameters (Å^2^)

**Table:**

	*x*	*y*	*z*	*U*_iso_*/*U*_eq_	
C1	0.3285 (4)	0.0505 (2)	0.7709 (4)	0.0525 (9)	
H1C	0.3682	0.0101	0.8405	0.063*	
H1D	0.3282	0.0188	0.6848	0.063*	
C2	0.4331 (4)	0.1290 (3)	0.7618 (4)	0.0518 (9)	
H2C	0.5384	0.1101	0.7346	0.062*	
H2D	0.4419	0.1571	0.8505	0.062*	
C3	0.2019 (3)	0.2241 (2)	0.6920 (3)	0.0373 (7)	
H3	0.2013	0.2560	0.7791	0.045*	
C4	0.0994 (4)	0.1433 (2)	0.7029 (4)	0.0429 (8)	
H4A	0.0917	0.1145	0.6147	0.051*	
H4B	−0.0066	0.1613	0.7297	0.051*	
C5	0.1468 (4)	0.2868 (2)	0.5815 (4)	0.0521 (9)	
H5A	0.1518	0.2569	0.4952	0.078*	
H5B	0.2141	0.3386	0.5801	0.078*	
H5C	0.0393	0.3047	0.5988	0.078*	
Cl1	0.20460 (10)	−0.02794 (6)	0.10506 (9)	0.0464 (2)	
Cl2	0.19838 (10)	0.21504 (6)	0.07287 (9)	0.0490 (2)	
Cl3	−0.04089 (10)	0.10987 (6)	0.33241 (9)	0.0470 (2)	
Cl4	0.40648 (12)	0.10940 (8)	0.37036 (10)	0.0670 (3)	
N1	0.1644 (3)	0.07835 (19)	0.8046 (3)	0.0428 (7)	
H1A	0.1639	0.1037	0.8877	0.051*	
H1B	0.1013	0.0297	0.8072	0.051*	
N2	0.3697 (3)	0.19527 (18)	0.6611 (3)	0.0379 (6)	
H2A	0.3721	0.1708	0.5775	0.046*	
H2B	0.4330	0.2438	0.6607	0.046*	
Zn1	0.19783 (4)	0.10028 (3)	0.22722 (4)	0.03895 (14)	

### Atomic displacement parameters (Å^2^)

**Table:**

	*U*^11^	*U*^22^	*U*^33^	*U*^12^	*U*^13^	*U*^23^
C1	0.056 (2)	0.043 (2)	0.059 (2)	0.0128 (17)	0.0036 (19)	0.0120 (17)
C2	0.0327 (18)	0.066 (2)	0.057 (2)	0.0041 (16)	−0.0075 (16)	0.0141 (19)
C3	0.0335 (16)	0.0407 (18)	0.0376 (17)	0.0001 (13)	0.0016 (14)	0.0058 (14)
C4	0.0302 (16)	0.050 (2)	0.049 (2)	−0.0042 (14)	0.0006 (14)	0.0125 (16)
C5	0.048 (2)	0.050 (2)	0.058 (2)	0.0058 (16)	−0.0006 (18)	0.0196 (18)
Cl1	0.0528 (5)	0.0424 (5)	0.0441 (5)	0.0075 (4)	0.0048 (4)	−0.0056 (4)
Cl2	0.0456 (5)	0.0490 (5)	0.0527 (5)	0.0104 (4)	0.0109 (4)	0.0079 (4)
Cl3	0.0396 (4)	0.0540 (5)	0.0474 (5)	0.0026 (4)	0.0088 (4)	−0.0057 (4)
Cl4	0.0453 (5)	0.1063 (9)	0.0493 (6)	0.0189 (5)	−0.0136 (4)	−0.0232 (5)
N1	0.0417 (15)	0.0416 (16)	0.0451 (16)	−0.0079 (12)	0.0057 (13)	0.0112 (13)
N2	0.0265 (13)	0.0475 (16)	0.0398 (15)	−0.0088 (11)	0.0010 (11)	0.0074 (12)
Zn1	0.0354 (2)	0.0457 (2)	0.0357 (2)	0.00775 (16)	−0.00171 (16)	−0.00409 (16)

### Geometric parameters (Å, °)

**Table:**

C1—C2	1.470 (5)	C4—H4B	0.9700
C1—N1	1.482 (4)	C5—H5A	0.9600
C1—H1C	0.9700	C5—H5B	0.9600
C1—H1D	0.9700	C5—H5C	0.9600
C2—N2	1.496 (4)	Cl1—Zn1	2.2616 (10)
C2—H2C	0.9700	Cl2—Zn1	2.2895 (10)
C2—H2D	0.9700	Cl3—Zn1	2.2702 (11)
C3—C4	1.487 (4)	Cl4—Zn1	2.2480 (12)
C3—C5	1.505 (4)	N1—H1A	0.9000
C3—N2	1.509 (4)	N1—H1B	0.9000
C3—H3	0.9800	N2—H2A	0.9000
C4—N1	1.495 (4)	N2—H2B	0.9000
C4—H4A	0.9700		
			
C2—C1—N1	110.4 (3)	C3—C5—H5A	109.5
C2—C1—H1C	109.6	C3—C5—H5B	109.5
N1—C1—H1C	109.6	H5A—C5—H5B	109.5
C2—C1—H1D	109.6	C3—C5—H5C	109.5
N1—C1—H1D	109.6	H5A—C5—H5C	109.5
H1C—C1—H1D	108.1	H5B—C5—H5C	109.5
C1—C2—N2	110.9 (3)	C1—N1—C4	111.8 (3)
C1—C2—H2C	109.5	C1—N1—H1A	109.3
N2—C2—H2C	109.5	C4—N1—H1A	109.3
C1—C2—H2D	109.5	C1—N1—H1B	109.3
N2—C2—H2D	109.5	C4—N1—H1B	109.3
H2C—C2—H2D	108.0	H1A—N1—H1B	107.9
C4—C3—C5	112.3 (3)	C2—N2—C3	112.7 (2)
C4—C3—N2	109.1 (3)	C2—N2—H2A	109.0
C5—C3—N2	108.5 (3)	C3—N2—H2A	109.0
C4—C3—H3	109.0	C2—N2—H2B	109.0
C5—C3—H3	109.0	C3—N2—H2B	109.0
N2—C3—H3	109.0	H2A—N2—H2B	107.8
C3—C4—N1	111.4 (3)	Cl4—Zn1—Cl1	111.20 (4)
C3—C4—H4A	109.3	Cl4—Zn1—Cl3	113.67 (4)
N1—C4—H4A	109.3	Cl1—Zn1—Cl3	108.70 (4)
C3—C4—H4B	109.3	Cl4—Zn1—Cl2	111.39 (5)
N1—C4—H4B	109.3	Cl1—Zn1—Cl2	106.39 (4)
H4A—C4—H4B	108.0	Cl3—Zn1—Cl2	105.07 (4)
			
N1—C1—C2—N2	−55.8 (4)	C3—C4—N1—C1	−57.4 (4)
C5—C3—C4—N1	175.0 (3)	C1—C2—N2—C3	55.8 (4)
N2—C3—C4—N1	54.6 (4)	C4—C3—N2—C2	−54.5 (4)
C2—C1—N1—C4	57.2 (4)	C5—C3—N2—C2	−177.2 (3)

### Hydrogen-bond geometry (Å, °)

**Table:**

*D*—H···*A*	*D*—H	H···*A*	*D*···*A*	*D*—H···*A*
N1—H1A···Cl2^i^	0.90	2.48	3.346 (3)	161
N1—H1B···Cl3^ii^	0.90	2.55	3.284 (3)	140
N1—H1B···Cl1^ii^	0.90	2.72	3.322 (3)	125
N2—H2A···Cl4	0.90	2.25	3.150 (3)	174
N2—H2B···Cl2^iii^	0.90	2.48	3.199 (3)	137
N2—H2B···Cl3^iii^	0.90	2.77	3.444 (3)	133

Symmetry codes: (i) *x*, *y*, *z*+1; (ii) −*x*, −*y*, −*z*+1; (iii) *x*+1/2, −*y*+1/2, *z*+1/2.
